# Supplementary material for: Association between oral microbiome and seven types of cancers in East Asian population: a two-sample Mendelian randomization analysis
Source: Front Mol Biosci. 2023 Nov 21;10:1327893. doi: 10.3389/fmolb.2023.1327893 (PMC10702768; doi:10.3389/fmolb.2023.1327893)
Supplement: Supplementary file 1 [file DataSheet1.ZIP › Supplementary Materials/MR plots of saliva/saliva═╝/Gastric cancer/pheno.3363_to_gastric cancer_forest.pdf]

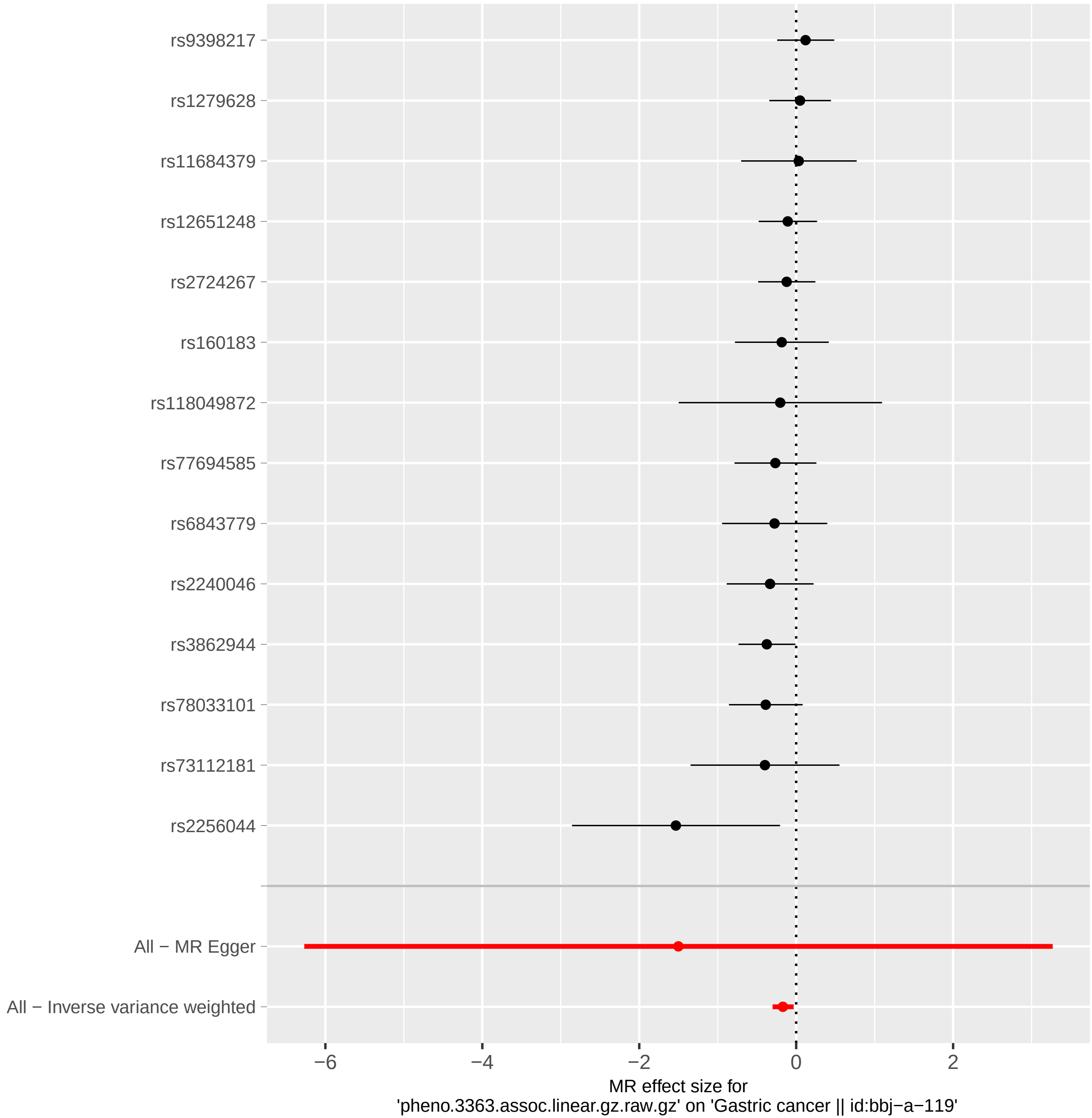

rs9398217
rs1279628
rs11684379
rs12651248
rs2724267
rs160183
rs118049872
rs77694585
rs6843779
rs2240046
rs3862944
rs78033101
rs73112181
rs2256044
All − MR Egger
All − Inverse variance weighted
−6
−4
−2
0
2
MR effect size for
'pheno.3363.assoc.linear.gz.raw.gz' on 'Gastric cancer || id:bbj−a−119'
